# Supplementary material for: Assessment of mucin-related gene alterations following treatment with rebamipide ophthalmic suspension in Sjögren’s syndrome-associated dry eyes
Source: PLoS One. 2020 Nov 24;15(11):e0242617. doi: 10.1371/journal.pone.0242617 (PMC7685436; doi:10.1371/journal.pone.0242617)
Supplement: Attachment — Submitted filename: response to reviewers.docx [file pone.0242617.s003.docx]

**Authors’ responses to the reviewers’ comments**

We express our sincere appreciation to the reviewers for their insightful comments, which have helped us to significantly improve our manuscript. We have addressed all comments below in a point-by-point manner (reviewers’ comments are shown in **bold-face** and italics).

**Reviewer #1**

***1. Some unusual data obatiend post-therapy at 12 weeks. I agree that the authors should investigate this in future studies by providing a diary or checklist to improve patient compliance. Is it possible that the efficacy of the drugs drops off in extended treatment periods?***

**Responses:** We agree with your supposition and believe that the reduction in therapeutic efficacy associated with long-term use of rebamipide ophthalmic suspension should be evaluated. For this paper, we have added a picture—Figure 6—of a representative patient in whom the treatment was efficacious. However, individual differences in the efficacy of rebamipide ophthalmic suspension is a question for future research. We have added the aforementioned information on lines 353–354 in the manuscript.

***2. Please check font size is consistent throughout text, as it varies somewhat.***

**Responses:** We appreciate your attention to detail. We have determined the font size according to the submission rules of PLOS ONE and standardized it throughout the manuscript.

***3. Do the authors have any photographs of cells on schirmer stripos (possible stained) to show how cells look in the 2 groups? Morphological assessments may be useful alongside mRNA data in explaining results observed.***

**Responses:** In impression cytology using filter paper (e.g., Shirmer’s test paper), it is difficult to examine the cells adhering to the filter paper by histological examination because the filter is stained by the staining fluid and does not become transparent. However, we believe that examining the type of cells collected by impression cytology is important to make clinical sense of our test results.

***4. No sex-matching was doen between controls and SS pateints. Can the authors briefly explain why they could not use all females to overcome this?***

**Responses:** We agree with your point; accordingly, we have revised the results to compare the SS-DE group with age- and sex-matched controls. We also revised Table 4 and Figure 5. Furthermore, we have revised the descriptions on Page 19, line 249–258.

***5. What was inclusion/exclusion criteria for controls? Only detail given for SS patients.***

**Responses:** The inclusion/exclusion criteria for the control group are described on Page 7, lines 90 to 92 as follows: “The control group consisted of 33 healthy adult volunteers (7 men and 26 women) without ocular disorders and who did not wear contact lenses.”

***6. What were the cut-off points used for TBUT for normals?***

**Responses:** The statement that the normal range for TBUT is longer than 5 seconds has been added on page 8, lines 117–118.

***7. Can authors explain why they stored mRNA at 4oc rather than at -20/-80 prior to extraction? RNA is notoriously unstable, even at -20.***

**Responses:** We placed the filter papers in RNAlater solution immediately after collecting the specimens. The mRNA extraction was performed on the day of sample collection. In preliminary experiments, we observed that mRNA extraction was better without repeated freeze-thaw cycles; therefore, it was stored at 4°C until extraction.

8. ***How did authors decide on the most appropriate control gene, GAPDH? Even control genes may be altered post-therapy (i.e. actin etc). Did they consider alternatives?***

**Responses:** We agree that this is an important point in performing quantitative PCR by the ΔΔCT method. GAPDH is a commonly used internal control. We compared the mRNA expression of actin with that of GAPDH, but there was no difference between the two. In the future, however, it would be desirable to create a calibration curve with a pre-determined standard mRNA.

***9. If previous mucin studies have sampled from the bulbal conjunctivae of SS-DE patients, why did the authors choose to sample from the upper palpebral conjunctivae?***

**Responses:** We performed impression cytology with filter paper as it has a lower specimen collection volume than with nitrocellulose membranes. Therefore, the upper palpebral conjunctiva was selected as the area where sufficient pressure could be applied to the filter paper. Future investigations are needed to examine optimal collection sites.

***10. Related to 9, usually SS patients are considered moderate-to-severe dry eye, due to the extended diagnosis times. Is this an early-diagnosis group to be considered mild-to-moderate? Or are more clinical assessments needed to confirm this diagnosis?***

**Responses:** This is an early-diagnosis group. As described in the Discussion section, systemic anti-inflammatory treatment for primary disease may have altered the severity of SS-DE (lines 317–319).

**Reviewer #2**

***1. The tile focuses on the “A method”, but actually the primary and secondary objective of this study focused on the mucin-related gene expression. I think the title should be considered to modify.***

**Responses:** We have changed the title as following: “Assessment of mucin-related gene changes following treatment with rebamipide ophthalmic suspension in Sjögren’s syndrome-associated dry eye.”

***2. The technique of impression cytology should be described more detailed including the position on the upper tarsal conjunctiva, the duration touched on it and how to press the paper. And usually the impression cytology is performed on the bulbar conjunctiva, why the authors choose the tarsal conjunctival?***

**Responses:** We have expanded the description about impression cytology as following: “the 5 mm tip of Schirmer’s test paper (Tear Production Measuring Strips, AYUMI Pharmaceutical Corporation, Tokyo, Japan) was placed at the center of the upper palpebral conjunctiva (Fig 1b) for 5 seconds using Beaupre cilia forceps” (pages 10, lines 138–139).

Impression cytology using filter paper is considered to have a lower specimen collection volume than that with nitrocellulose membranes. Therefore, we selected the upper palpebral conjunctiva as the area where sufficient pressure could be applied to the filter paper. Furthermore, we felt that the upper palpebral conjunctiva was suitable for the investigation of mucin-related genes because this region has a high density of goblet cells. However, in the present study, these features may have caused the difference between the control and SS-DE groups to be less pronounced.

***3. Did the SS-DE patients use only rebamipide during this study? The authors should state it clearly. This may associate with the unusual change of the data in week 12.***

**Responses:** We agree with your suggestion and, accordingly, have added following description:

“The rebamipide eye drops were supplemented with sodium hyaluronate in three patients, and with artificial tears and sodium hyaluronate in one; no additional medications were added for nine patients. The compositions of the eye drop were not changed during the observation period.” (page 15, lines 205–208).

***4. The data of this study showed significantly difference of mucin mRNA expression between the male and female controls. So, I think when compares the baseline mucin level, the authors can only use the female data in the control group to compare with SS group, because there is no male in SS group. Current results showed there was no difference of the mucin expression between controls and the baseline of SS group which was not consistent with most studies. When the authors removed the influence of gender, the results may change.***

**Responses:** We have revised the results to comparing the SS-DE group with age- and sex-matched controls. We have also revised Table 4 and Figure 5. Furthermore, we have revised the descriptions on Page 19, line 249–258.

***5. It will be better if the authors could provide representative fluorescein staining photos before and after treatment.***

**Responses:** We have added Figure 6, which shows the eyes of a representative case.
